# Comprehensive overview of Nrf2-related epigenetic regulations involved in ischemia-reperfusion injury

**DOI:** 10.7150/thno.77243

**Published:** 2022-09-11

**Authors:** Jun Zhang, Wanqian Pan, Yue Zhang, Mingyue Tan, Yunfei Yin, Yuanmei Li, Lei Zhang, Lianhua Han, Jiaxiang Bai, Tingbo Jiang, Hongxia Li

**Affiliations:** 1 Department of Cardiology, The First Affiliated Hospital of Soochow University, 188 Shizi Street, Suzhou, Jiangsu 215006, China.; 2 Department of Orthopedics, The First Affiliated Hospital of Soochow University, Suzhou 215000, China.; 3 Department of Cardiology, Kunshan Hospital of Traditional Chinese Medicine, Kunshan Affiliated Hospital of Nanjing University of Chinese Medicine, Suzhou 215000, Jiangsu, China.

**Keywords:** epigenetic modifications, ischemic-reperfusion injury, Nrf2, oxidative stress, therapy

## Abstract

Ischemic disease is a class of diseases in which an organ is ischemic due to vascular occlusion, a major contributor to death and disability worldwide. However, when the blood flow is restored, more severe damage occurs than ischemia alone and is known as ischemic-reperfusion injury (IRI). During reperfusion, the imbalance between the production of reactive oxygen species (ROS) and buffering capacity of the antioxidant defense system results in cell damage and death. Nuclear factor E2-related factor 2 (Nrf2) significantly affects antioxidant stress damage. The function of Nrf2 in the pathological process of IRI has been widely discussed, but the impact of epigenetic modifications associated with Nrf2 remains unclear. This article provides a comprehensive overview of the role and mechanism of Nrf2-related epigenetic modifications in the IRI of various organs, including the brain, heart, liver, and kidney. In addition, we summarize agonists that may target epigenetic regulation of Nrf2, which may be beneficial in seeking more effective strategies to improve IRI.

## Introduction

Many diseases lead to impaired blood circulation, which can contribute to ischemia in various organs, including the brain, heart, liver, and kidneys 1. Prolonged ischemia of an organ results in irreversible damage. Blood flow restoration by clinical intervention is the major treatment strategy for ischemic organs, known as reperfusion therapy, which can effectively reduce tissue damage and ameliorate patient mortality. However, reperfusion causes additional damage creating a significant challenge in the current treatment of ischemic disease 2, 3.

The role of oxidative stress, inflammation, and apoptosis in the initiation and development of ischemic-reperfusion injury (IRI) has been extensively studied 4, 5. It is noteworthy that oxidative stress is perceived as a primary contributor to IRI. The continuous production of oxidative and electrophilic chemicals irreversibly damages cellular lipids, proteins, and nucleic acids 6. A complex antioxidant defense system has evolved to counteract the stress generated by endogenous and exogenous oxidants and electrophiles that includes nuclear factor erythroid 2-related factor 2 (Nrf2), protein kinase C (PKC), protein kinase B (PKB), and hypoxia-inducible factor-alpha (HIF-α). Among them, Nrf2, encoded by *Nfe2l2*, is considered to be a master regulator that protects cells from the effect of oxidative stress 7, 8. Nrf2 activities differ greatly depending on the stage of various pathological processes. Although somatic mutations or single nucleotide polymorphisms (SNPs) could explain this difference, somatic mutations are only present in a small subset of tissues 9-11, indicating the existence of other mechanisms regulating Nrf2 expression.

Epigenetic modifications refer to heritable changes in the phenotype of cells, principally involving DNA methylation, histone modifications, and noncoding RNAs and, are not affected by changes in the DNA sequence. There is substantial evidence that epigenetic mechanisms can modulate Nrf2 expression in cancer and other diseases 7, but the application of epigenetics to IRI is still a relatively young field rapidly gaining attention. With improved research methods, key advances have been made, contributing to the progress of this field. Recently, hypermethylation of CpG islands in the promoter region of the Nrf2 gene was found to reduce Nrf2 expression and increase oxidative stress, inflammation, and apoptosis 12. Acetylation, methylation, phosphorylation, and ubiquitination are the amino terminus modification of histones, among which acetylation and methylation have a dominant role in IRI by affecting the Nrf2 gene expression 13. Moreover, modulation of the Nrf2 gene by noncoding RNAs has also been investigated in IRI 14, 15. Unlike genetic mutations, epigenetic changes are reversible and susceptible to environmental factors. Therefore, developing drugs targeting epigenetic mechanisms represents a potential direction for clinical research.

Currently, preclinical studies and clinical trials are extensively employed to validate effective therapeutic strategies. From this perspective, it is necessary to understand the underlying cellular signaling pathways relevant to the development and treatment of IRI. Herein, we review the structure of Kelch-like ECH-associated protein 1 (Keap1)/Nrf2 and their epigenetic mechanisms, focusing on their functions in oxidative stress, apoptosis, inflammation, and endoplasmic reticulum stress during IRI in various organs, including the brain, heart, liver, and kidney. Furthermore, we summarize recent advances in the pharmacotherapy of Nrf2-related epigenetic modifications that might help prevent IRI.

## Structures and interactions of Keap1/Nrf2

**Figure 1:**
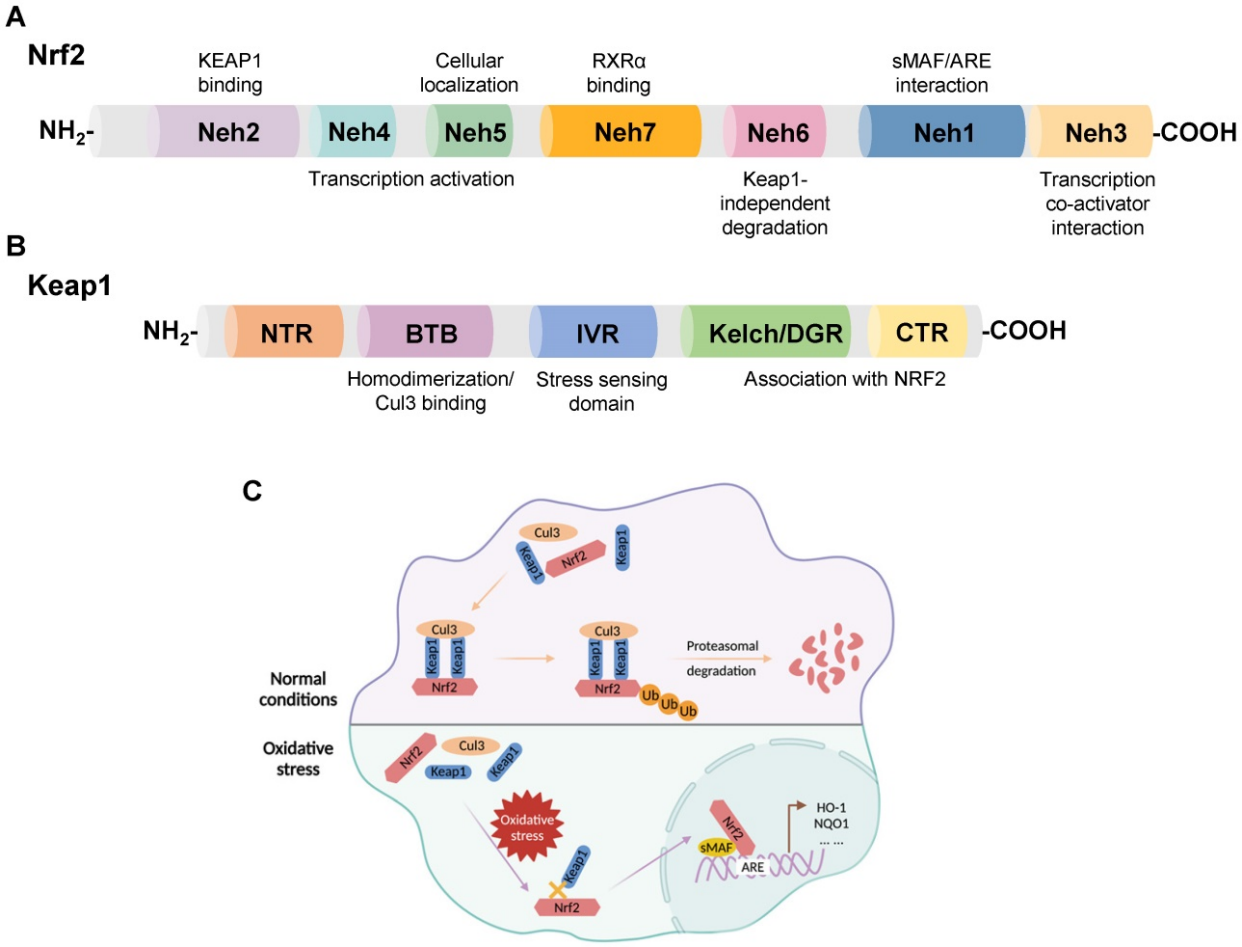
** (A)** Domain structures of Nrf2. **(B)** Domain structures of Keap1. **(C)** Regulatory mechanism of Keap1 on the cellular abundance of Nrf2. Under physiological conditions, Keap1 serves as a link between Nrf2 and Cul3, promoting the rapid degradation of Nrf2 in the cytoplasm through the ubiquitin-proteasome pathway. When cells suffer oxidative stress damage, the Keap1-Nrf2 interaction is disrupted, and Nrf2 translocates to the nucleus, forming a heterodimerization complex with sMAF. Nrf2-sMAf heterodimers recognize ARE sequences and activate the transcription of antioxidant genes, such as HO-1 and NQO1.

Nrf2, a member of the Cap-n-Collar family of basic leucine zipper proteins, was initially identified as an activator of β-globin gene expression and later shown to be a key sensor of cellular oxidative stress 16, 17. Nrf2 is widely expressed in oxygen-consuming organs, such as the heart, liver, brain, kidney, muscle, and blood vessels. Nrf2 consists of 605 amino acids with seven functional domains, known as Nrf2-ECH homology (Neh) 1-7, responsible for preserving the stability of Nrf2 and modulating its transcriptional activity 18-20. Among these seven domains, Neh1, 3, 4, and 5 are associated with transcriptional activation, while Neh2, 6, and 7 are negative regulatory domains 21. In addition, Nrf2, as a transcription factor, is instrumental in maintaining redox homeostasis by inducing the expression of numerous genes involved in antioxidant defense, such as nicotinamide adenine dinucleotide phosphate (NADPH), glutathione peroxidase (GPx), NQO1, HO-1, and ferritin 22-26 (**Figure 1A**).

Keap1, a 624 amino acid protein, is an adapter for the Cul3-Rbx E3 ubiquitin ligase complex with five domains: an N-terminal region (NTR); a Broad complex, Tramtrack, and Bric-á-Brac (BTB) domain; an intervening region (IVR); a double glycine repeat (DGR) and a Kelch domain; the C-terminal region (CTR) 27, 28. DGR and CTR mediate the binding between Keap1 and Nrf2. The BTB domain functions by boosting the homodimerization of Keap1 and facilitating the link between Keap1 and Cul3 6 (**Figure 1B**).

Nrf2 is degraded by its negative regulator, Keap1. In physiological states, Keap1 acts as a link between Nrf2 and the ubiquitination ligase Cullin-3 (Cul3), promoting the rapid degradation of Nrf2 in the cytoplasm through the ubiquitin-proteasome pathway. When subjected to oxidative stress, the interaction between Keap1 and Nrf2 is impaired, and Nrf2 translocates from the cytoplasm to the nucleus. Subsequently, Nrf2 forms a heterodimerization complex with the transcriptional regulator musculoaponeurotic fibrosarcoma (sMAF), which recognizes antioxidant response elements (AREs) and promotes the transcription of antioxidant genes, such as heme oxygenase-1 (HO-1), NAD(P)H quinone oxidoreductase 1 (NQO1), and glutathione-s-transferase (GST) 29, 30 (**Figure 1C**).

The expression and activity of Keap1 and Nrf2 are affected by various factors. Currently, the focus is on regulating their expression and activity at the transcriptional level through epigenetic modifications, providing new ideas for the application of Keap1/Nrf2 in disease treatment.

## Epigenetic modifications

**Figure 2:**
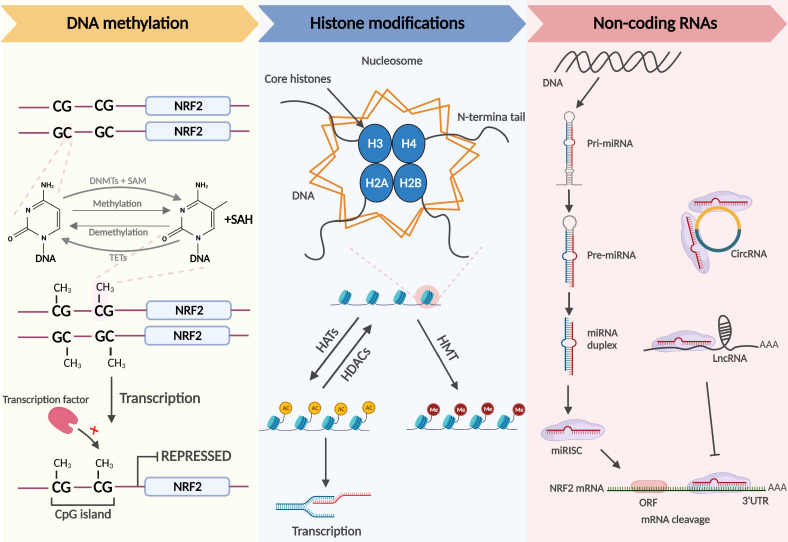
** Nrf2-related epigenetic modifications: DNA methylation.** DNMTs methylate the C5 position of cytosine in DNA to generate 5-methylcytosine, which is progressively oxidized to 5-carboxylcytosine by TETs. When the CpG islands in the promoter region are extensively methylated, the transcription factor cannot identify the promoter, and Nrf2 transcription is repressed. **Histone modifications.** Chromatin consists of DNA and histone complexes. In each nucleosome, four core histone proteins (H2A, H2B, H3, H4) form an octamer around which DNA is tightly wrapped. Histone tails, the free amino ends of histones, can be modified differently under the action of related enzymes, affecting gene expression. Histone acetylation and deacetylation are regulated by HATs and HDACs and hyperacetylated histones are associated with transcriptional activation. Histone tail lysine and arginine residues are methylated by HMTs. The effect of histone methylation on transcription depends on the methylation site and the methylation stoichiometry. **Noncoding RNAs.** Mature miRNAs identify the complementary bases of the 3'UTR in the Nrf2 mRNA and trigger mRNA cleavage. CircRNAs and lncRNAs contain miRNA binding sites that act as competing endogenous RNAs (ceRNAs) to regulate the expression of miRNA target mRNAs.

The term epigenetics was originally used to describe the complex mutual effects between the genome and the environment involved in the development and differentiation of higher organisms 31. Epigenetics is currently used to explain the phenomenon in which gene expression alterations occur without changes to the nucleotide sequence 32. Similar to genetic factors, it preserves cell differentiation and mammalian development 33. The most common epigenetic alterations are DNA methylation, histone modifications, and the regulation of noncoding RNAs, which modulate cell differentiation, cell-specific gene expression, and genome stability and structure 34 (**Figure 2**).

DNA methylation, the transfer of methyl groups onto the C5 position of cytosine, occurs mainly in CpG-rich regions called CpG islands that are present in regulatory regions, such as promoters or enhancers of genes 35. When CpG islands in the promoter region are heavily methylated, it is difficult for transcription factors to recognize the promoter, and gene expression is shut down. DNA methyltransferases (DNMTs) include DNMT3a, DNMT3b, and DNMT1. DNMT3a and DNMT3b, known as de novo DNA methyltransferases, are methylate double-stranded DNA that have never been methylated. Usually, the nucleosome DNA is the substrate of de novo DNA methyltransferases, and modifications of histones in nucleosomes affect the ability of these enzymes for de novo methylation. DNMT1 maintains DNA methylation and can methylate cytosine to methylcytosine in the double strands of semi-methylated DNA molecules, participating in the methylation of new synthetic chains in DNA replication 35, 36. Demethylation of DNA boosts subsequent gene expression. Currently, there are two generally accepted methods of DNA demethylation, namely, active and passive demethylation. The former refers to the oxidation of methyl groups to hydroxymethylation that is then repaired in the presence of ten-eleven translocation (TET) methylcytosine dioxygenases; the latter partly occurs in the case of low DNMT activity or a low amount of methyl donor during replication. In addition, passive demethylation may also arise when methylated cytosine is oxidized by TET enzymes to hydroxymethyl-cytosine, which cannot be distinguished by DNMT1, resulting in the unmethylated cytosine on the synthetic chains 34.

Chromatin, the basic structural units of which are nucleosomes, consists of macromolecular DNA and histone complexes. In each nucleosome, four core histone proteins (H2A, H2B, H3, H4) form an octamer around which DNA is tightly wrapped. The free amino ends of histones in the core region of the nucleosome are called histone tails, which can be modified differently by enzymes and affect the transcriptional activity of genes. Post-translational modifications in histone tails consist of acetylation, methylation, ubiquitination, and phosphorylation. Histone acetylation is modulated by histone acetyltransferases (HATs) and histone deacetylases (HDACs). HATs transfer acetyl groups to lysine residues to neutralize the positive charge in histones. Because DNA is negatively charged, increased acetylation results in a weak interaction between histones and DNA, prompting nucleosomes to become loose and enabling transcription. Thus, histone acetylation is usually associated with transcriptional activation, while deacetylation participates in derailing gene expression 37.

Histone methylation refers to the transfer of methyl groups to the histone tail by histone methyltransferases (HMTs) and usually occurs on arginine or lysine residues. The regulatory role of histone methylation on transcription depends on the modification site and the methylation stoichiometry (monomethylation, dimethylation, or trimethylation) 38. Another epigenetic modification, histone ubiquitination 39, is the process of the sequential actions of E1 activating, E2 conjugating, and E3 ligase enzymes, resulting in the covalent conjugation of ubiquitin, a 76 amino acid protein, to a lysine residue on proteins, which plays a key role in transcription initiation and elongation, silencing, and chromatin structure 40, 41. Ubiquitination can be reversed by deubiquitinases (DUBs). DUBs hydrolyze ester bonds, peptide bonds, or isopeptide bonds at the carboxyl terminus of ubiquitin, specifically separating ubiquitin from protein substrates and regulating deubiquitination.

Noncoding RNAs, including microRNAs (miRNAs), long noncoding RNAs (lncRNAs), and circular RNAs (circRNAs), are functional RNA molecules that cannot be translated into proteins but have been identified as epigenetic regulators, playing a significant role in controlling Nrf2 gene expression 42, 43. MiRNAs are composed of 18-25 nucleotides and serve as post-transcriptional regulators of gene expression, can bind to the untranslated regions (UTRs) of mRNA, and this imperfect sequence complementarity contributes to translation inhibition and/or mRNA degradation. Moreover, the expression of miRNAs is also modulated by epigenetic mechanisms 44, 45. CircRNAs have a stable circular structure that prevents exonuclease-mediated elimination. LncRNAs are large noncoding RNAs > 200 nucleotides in length. Recent studies have shown that circRNAs and lncRNAs hold rich miRNA binding sites that can act as miRNA sponges, indirectly modulating the expression of targeted genes 46.

The effect of epigenetic modifications on the Keap1/Nrf2 signaling pathway has been demonstrated. In the following section, we discuss the mechanisms of Keap1/Nrf2-related epigenetic modifications and beneficial therapeutic agents in IRI.

## Nrf2-related epigenetic modifications in the pathological process

### Oxidative stress

**Figure 3:**
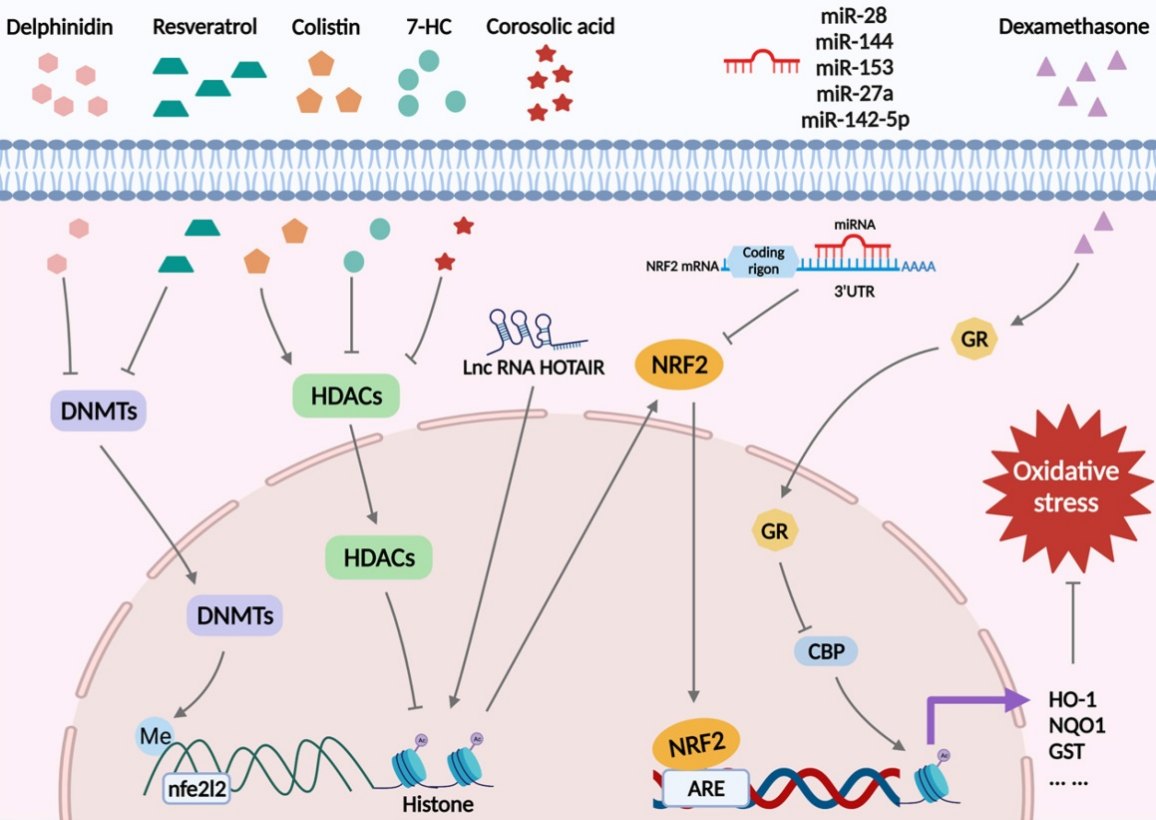
**Nrf2-related epigenetic modifications associated with oxidative stress.** Delphinidin and resveratrol play a role in antioxidant reactions by decreasing DNMTs and reducing DNA methylation in the *Nfe2l2* promoter region. Colistin reduces the acetylation of histones by inducing HDAC expression and reducing Nrf2. 7-HC and corosolic acid increase histones acetylation to exert antioxidant effects by decreasing HDAC expression. LncRNA HOTAIR increases H4 acetylation on the *Nfe2l2* promoter, which, in turn, promotes Nrf2 expression. MiR-28, miR-144, miR-153, miR-27a, and miR-142-5p promote mRNA degradation by binding to the 3'UTR of Nrf2 mRNA, inhibiting Nrf2 expression and increasing oxidative stress. Dexamethasone inhibits the expression of Nrf2 target antioxidant genes by enhancing GR enrichment to AREs and blocking CBP recruitment and histone acetylation at AREs.

Oxidative stress arises when there is an imbalance between the oxidant and antioxidant defense systems of the body 8. This imbalance results in apoptosis, autophagy, inflammation, and other damage to cells via various signaling pathways, resulting in irreversible cell injury and organ dysfunction 20, 47. Nrf2 is known as the “master regulator” of the antioxidant process, responsible for modulating the antioxidant system and the expression of cell-protective genes 48-50. Nrf2 is widely present in oxygen-depleting organs such as the brain, heart, liver, kidneys, and blood vessels. Due to its positive impact on oxidative stress damage, Nrf2 has protective effects on multiple diseases. For example, it has been reported to protect nerve cells from IRI 51, and etomidate, an anesthetic agent, exerts a beneficial effect on myocardial tissue by activating the Nrf2/ARE signaling pathway 52. Thus, it is necessary to study the mechanism of action of Nrf2 in redox imbalance. In this section, we will summarize the specific mechanisms of Keap1/Nrf2 signaling in oxidative stress from the epigenetic modification perspective (Figure 3).

DNA demethylation of the Keap1 promoter CpG islands activates Keap1 protein production in the lens of diabetic cataract patients, increasing Nrf2 proteasomal degradation, altering lens protein redox balance, and blocking the cellular response to stress 53. In addition, in 12-O-tetradecanoylphorbol-13-acetate (TPA)-induced mouse skin epidermal JB6 P_+_ cells, decreased DNA methylation in the first 15 CpG sites in the *Nfe2l2* promoter region by delphinidin could alleviate oxidative stress injury by restraining DNMT1 and DNMT3a 54. Also, a recent study showed downregulated Nrf2 expression in a mouse prostate cancer model, and mechanistic studies identified a CpG island in the Nrf2 promoter region in tumorigenic TRAMP C1 cells but not in normal prostate or non-tumorigenic TRAMP C3 cells. Methylation of these CpG islands significantly repressed Nrf2 transcription in TRAMP C1 cells and augmented the recruitment of methyl-CpG-binding protein 2 (MBD2) and trimethyl-histone H3 (Lys9) proteins to the CpG islands in TRAMP C1 cells compared with TRAMP C3 cells. In contrast, the binding of RNA Pol II and acetylated histone H3 to the Nrf2 promoter was reduced. Intervention with 5-aza-29-deoxycytidine (5-aza), which inhibits DNMTs, and trichostatin A (TSA), an HDAC inhibitor, the expression of Nrf2 and NQO1 was restored in TRAMP C1 cells, verifying that hypermethylation of five CpG sites at the Nrf2 promoter was associated with mouse prostate cancer 55. A similar phenomenon was observed in human prostate cancer tissues, where three specific CpG sites in the Nrf2 promoter were hypermethylated 56. Interestingly, in mouse skin epidermal JB6 P_+_ cells, the flavonoid taxifolin showed the same effect 57. Furthermore, methylation of the Nrf2 promoter region was upregulated in high glucose (HG)-treated HepG2 cells, and resveratrol restricted HG-induced ROS production through demethylation of the Nrf2/ARE signaling pathway 58. Conversely, the combined therapeutics of trastuzumab and pertuzumab promoted DNA methylation on the *Nfe2l2* promoter in human ovarian cancer cells, weakening the expression of Nrf2 and attenuating its antioxidant capacity, thereby achieving an anticancer effect 59.

Recently, Wang et al. showed that in kidney cells, colistin significantly diminished the expression of HDAC1 and deacetylated histone 3 in the Nrf2 promoter region at Lys27, inhibiting Nrf2 signaling, increasing cellular oxidative stress and apoptosis, and leading to renal damage. The coumarin derivative, 7-Hydroxycoumarin (7-HC) could attenuate HDAC1 level and restore histone acetylation in the Nrf2 promoter region to increase Nrf2 expression, thus enhancing the antioxidant capacity of the kidney 60. A study using TRAMP C1 prostate cells indicated that corosolic acid treatment promoted histone H3 lysine 27 (H3K27ac) acetylation while reducing the trimethylation of histone H3 lysine 27 (H3K27me3) in the promoter region of Nrf2, which might be related to a decrease in HDACs 61. Furthermore, another study showed that in mouse hepatocellular carcinoma (Hepalclc7) cells, dexamethasone facilitated glucocorticoid receptor (GR) recruitment to AREs, inhibiting the assembly of Nrf2-dependent cAMP-response element-binding protein (CBP) and histone acetylation at AREs and the subsequent transcription of downstream Nrf2, blocking its antioxidant capacity 62.

In reticulocytes of patients with sickle cell disease, miR-144 downregulated Nrf2 expression, thereby impairing tolerance to oxidative stress 63. Similarly, miR153/miR27a/miR142-5p/miR144 reduced Nrf2 expression by targeting *Nfe2l2* and increased the sensitivity of neuronal SH-SY5Y cells to oxidative stress 64. Yang et al. reported that miR-28 aggravated oxidative stress in breast epithelial cells by targeting the 3′UTR of Nrf2 mRNA 65. Dioscin, a natural steroid saponin, was shown to activate Nrf2, Sirtuin 2 (SIRT2), and downstream antioxidant stress-related target genes, such as glutamate-cysteine ligase modifier subunit (GCLM), forkhead box O3 (FOXO3a), HO-1, and NQO1 in H9C2 cells by significantly reducing the expression level of miR-140-5p 66. Moreover, Hox transcript antisense intergenic RNA (HOTAIR) was the first lncRNA documented to modulate Nrf2 expression. In the sperms of asthenozoospermic and oligoasthenozoospermic patients, HOTAIR reduction attenuated the ability to resist oxidative stress by limiting the acetylation of H4 in the *Nfe2l2* promoter, which in turn impeded Nrf2 expression 66.

### Inflammation

Inflammation underlies various physiological and pathological processes triggered by harmful stimuli, such as infection and tissue damage. In addition, oxidative stress can also promote inflammatory responses. In addition, oxidative stress can also promote inflammatory responses. Cells damaged by ROS release damage-related model molecules (DAMPs), which combine with TLR4 or TLR9 on KCs to activate the NF-kB signaling pathway and thus generate more ROS to amplify the inflammatory response 67, 68. Epigenetic modifications have been reported to be involved in the inflammatory process, and numerous studies have focused on the effect of epigenetic modifications on Nrf2 during the inflammatory response. One recent study found that environmental enrichment increased DNA methylation and histone H3 and H4 acetylation levels in the SAMP8 mouse hippocampus, likely associated with increased Nrf2 expression and decreased expressions of inflammatory genes, such as IL-6 and Cxcl10 69. Significant differences in Nrf2 and histone deacetylase expression were also observed in patients with type 2 diabetes and diabetic foot ulcers compared with healthy controls. Another study found a significantly positive correlation of HDAC4 with IL-6, and sirtuin 1 (SIRT1) was negatively correlated with IL-6, indicating that in the Nrf2 signaling pathway, HDAC4 and SIRT1 had pro-inflammatory anti-inflammatory effects, respectively 70. Huang et al. reported that HDAC3 inhibition could promote anti-inflammatory gene expression through the Keap1-Nrf2-Nox4 signaling pathway and improve endothelial dysfunction in type 2 diabetes mellitus 71. Interestingly, Nrf2 could also regulate HDAC expression; in mice exposed to cigarette smoke, deletion of the Nrf2 gene resulted in decreased HDAC2 and increased inflammation in the lung tissue 72.

### Apoptosis

Apoptosis, a mode of programmed cell death, plays a significant role in the development of various diseases. Physiologically, apoptosis helps the body remove abnormal and harmful cells. In pathological conditions, injury-induced excessive apoptosis will aggravate tissue damage. Therefore, it is extremely important to study the role of apoptosis and the regulation of apoptosis in diseases. Recently, the regulation of apoptosis by Nrf2-related epigenetic modifications has attracted much attention. In the human lung cancer cell line H292, licochalcone A, a natural flavonoid, could increase endoplasmic reticulum stress and apoptosis by increasing miR-144-3p and downregulating Nrf2 73. A commonly used anesthetic agent, sevoflurane inhibited histone methyltransferase G9a and histone H3 lysine 9 (H3K9me2), promoted Nrf2 expression, and reduced the levels of inflammatory factors and neuronal apoptosis, attenuating hypoxic-ischemic brain injury in neonatal rats 74. In podocytes, overexpression of Sirt6, a histone deacetylase, could increase the expression of Nrf2 and HO-1 genes and inhibit angiotensin (Ang) II-induced ROS generation and DNA double-strand breaks, reducing Ang II-induced podocyte apoptosis 75. Also, Shao et al. found that in Pc12 cells, overexpression of miR-153 promoted the Nrf2/ARE signaling pathway, which inhibited neuronal apoptosis and promoted cell growth, alleviating isoflurane-induced neurotoxicity 76.

## Nrf2-related epigenetic modifications in IRI

IRI is a pathological event that occurs in various diseases, causing severe cellular damage and death. As the name implies, IRI includes two distinct stages. Ischemia, the blockage of the arterial blood supply to an organ by emboli, is the first significant stage, while the second predominant process is reperfusion, the restoration of blood flow and oxygen supply to the affected ischemic area, which aggravates oxidative stress injury and contributes to excessive tissue deterioration. IRI can occur in many organs, such as the heart, brain, kidneys, lungs, and muscle 77, 78. Besides the ischemic organs, IRI can cause systemic damage and negatively affect other organs, leading to multiple organ failure 79. There is an imperative need to explore new therapeutic approaches and effective treatments. Nrf2 plays an important role in IRI due to its extensive biological effects, including anti-oxidative stress, anti-inflammation, and improved mitochondrial function. Recently, emerging evidence has indicated that the epigenetic modification of Nrf2 is involved in the pathophysiology of IRI. In this section, we discuss the latest research progress on Nrf2-related epigenetic mechanisms in ischemia-reperfusion (I/R) diseases focusing on the nervous, circulatory, digestive, and urinary systems.

### Cerebral IRI

**Figure 4:**
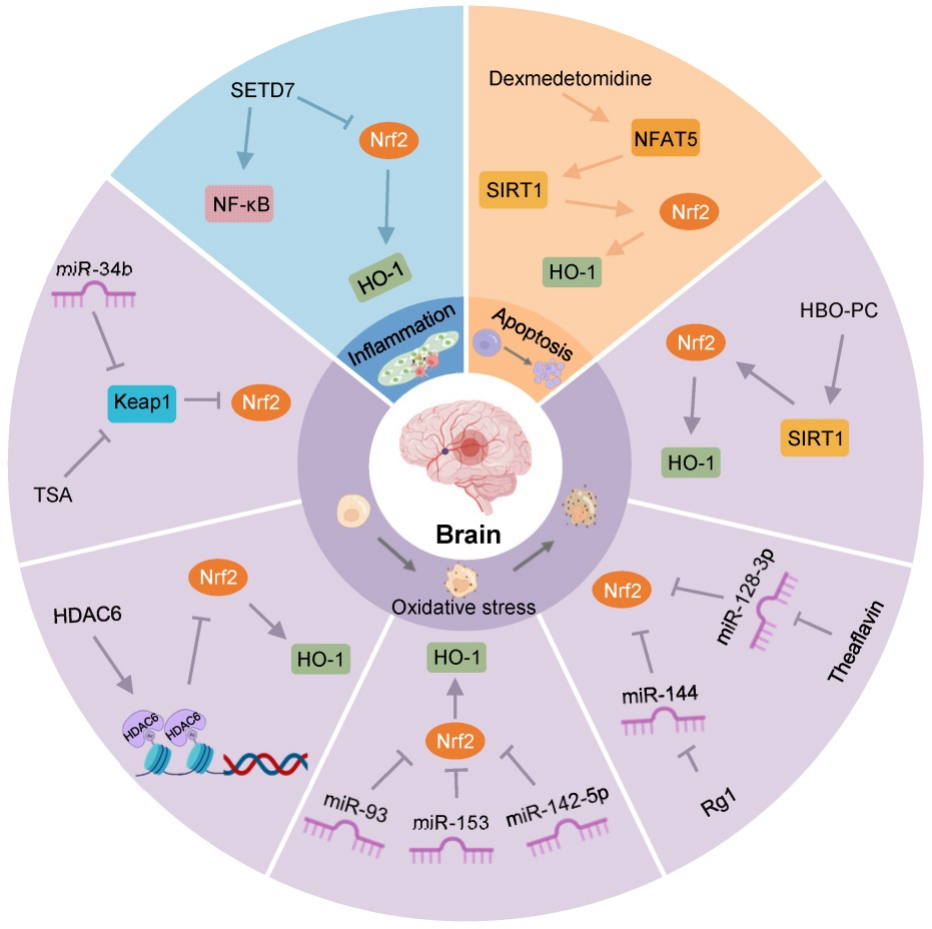
** Nrf2-related epigenetic modifications in cerebral IRI.** TSA increases Nrf2 and its nuclear translocation by decreasing Keap1 levels, and reduces oxidative stress after OGD. MiR-34b increases Nrf2 by decreasing Keap1 to relieve oxidative stress in cerebral IRI. HDAC6 can promote histone deacetylation, reducing the expression of Nrf2. MiR-93, miR-153, and miR-142-5p might restrain the Nrf2/HO-1 pathway to exert an antioxidant effect on OGD/R-induced neurocyte injury. Rg1 may promote Nrf2 expression by restraining miR-144 to protect against I/R-induced oxidative stress damage. Theaflavin activates Nrf2 by restraining miR-128-3p to resist oxidative stress in cerebral IRI. HBO-PC alleviates oxidative stress damage in rats by promoting the SIRT1/Nrf2/HO-1 axis. Activation of the NFAT5/SIRT1/Nrf2 pathway by dexmedetomidine may reduce apoptosis in diabetic cerebral IRI. SETD7 aggravates OGD/R-induced inflammatory responses by inhibiting Nrf2 and activating NF-κB.

Stroke is the second leading cause of death and one of the major drivers of long-term disability globally 80. Although various treatment methods restore blood flow in the infarcted area, the prognosis of patients has not been significantly improved, mainly due to IRI. Cerebral IRI often occurs after a clinical stroke, myocardial infarction, and organ transplantation. Ischemia and hypoxia cause neuronal impairment, necrosis, brain tissue damage, and metabolic dysfunction, which are further aggravated after blood flow is restored. The physiological and pathological mechanisms are complex and involve oxidative stress, excessive activation of inflammatory responses, and apoptosis or necrosis 81. In IRI, regulating the expression of Keap1/Nrf2 through epigenetic modification can affect its downstream targets to alleviate brain tissue injury (**Figure 4**). Therefore, this section aims to investigate the role of epigenetic modifications of Keap1/Nrf2 in ameliorating cerebral IRI, paving the way for the development of new therapeutic approaches.

Recently, Li et al. observed that the expression of HDAC6, a class IIB HDAC isoenzyme, was increased in the brain tissue of cerebral I/R mice, while Nrf2 content was significantly decreased in the nucleus. Further research found that HDAC6 interference could reduce the infarct size of brain tissue and improve neurological function by considerably increasing the expression levels of Nrf2 and HO-1 13. Similarly, another study showed that HDAC6 inhibition could reduce oxidative stress damage and exert neuroprotective effects by activating Nrf2 82.

SIRT1 is an NAD^+^-dependent class III histone deacetylase that regulates the acetylation of specific transcription factors and proteins 83. For example, in cerebral I/R rats subjected to hyperbaric oxygen preconditioning (HBO-PC), increased SIRT1 promoted the expression of Nrf2, HO-1, and superoxide dismutase 1 (SOD1), lowering oxidative stress damage and playing a neuroprotective role in transient focal cerebral ischemia. At the same time, this protective effect was inhibited by SIRT1 or Nrf2 interference 84, suggesting that SIRT1, as a key epigenetic regulator, plays a significant role in ameliorating cerebral IRI by regulating the expression of Nrf2.

In oxygen-glucose-serum deprivation/restoration (OGSD/R)-treated astrocytes, overexpression of the nuclear factor of activated T cells 5 (NFAT5), a member of the Rel family, could significantly decrease histone acetylation and promote Nrf2 nuclear transport to reduce oxidative stress and apoptosis 85. Additional studies have reported that dexmedetomidine attenuated oxidative stress, inflammation, and apoptosis via the NFAT5/SIRT1/Nrf2 signaling pathway, thus protecting the brain from the effects of IRI aggravated by diabetic hyperglycemia 86.

The protein SET domain-containing lysine methyltransferase 7 (SETD7), a class of lysine methyltransferases, has been identified as a key enzyme in the lysine methylation of histone and non-histone proteins 87-89. Using an *in vitro* cell model of cerebral IRI, Pan et al. observed increased SETD7 expression in cells, decreased PC12 cell viability, and increased apoptosis. However, SETD7 knockout could reverse the effects of oxygen-glucose deprivation/reoxygenation (OGD/R) on PC12 cell viability and apoptosis by increasing Nrf2 expression and inactivating NF-κB, simultaneously diminishing the inflammatory response and oxidative stress 90.

TSA increases neuron viability and reduces infarct size in stroke mice after OGD. *In vitro* studies revealed that TSA reduced Keap1 level and induced Keap1/Nrf2 dissociation and Nrf2 nuclear translocation. Subsequently, Nrf2, combined with the ARE in the HO-1 gene, triggered HO-1 transcription, exerting a protective effect on hypoxic neurons 91.

MiRNAs also regulate neuronal survival during cerebral IRI. For example, in hippocampal neurons, miR-142-5p was involved in OGSD/R-induced cellular damage, and its downregulation relieved oxidative stress and neuronal damage by promoting Nrf2 expression 92. Ji et al. reported that miR-153 could degrade Nrf2 in neurons, and its downregulation could promote the expression of Nrf2 and HO-1, alleviating OGSD/R-induced neuronal lesions and oxidative stress 93. MiR-34b was predicted to limit Keap1 expression by binding to the 3'-UTR of Keap1 in rats. After focal I/R of the cerebrum, miR-34b expression was time-dependently downregulated. The overexpression of miR-34b increased Nrf2 and HO-1 protein levels to rectify I/R-induced oxidative stress damage and the volume of cerebral infarction by directly restraining Keap1 expression 94. Theaflavin, derived from black tea, significantly reduced I/R-induced neuronal damage by eliminating miRNA-128-3p-mediated Nrf2 inhibition and oxidative stress, thereby improving impaired memory and learning ability 95.

Furthermore, ginsenoside Rg1, a saponin extracted from Panax ginseng, has been shown to protect against I/R-induced oxidative stress through the miR-144/Nrf2/ARE pathway. Specifically, in OGSD/R-treated PC12 cells, Rg1 markedly decreased miR-144, downregulating Nrf2 production by targeting its 3′-UTR 15. Similarly, miR-93 was increased in brain tissue after ischemia and could directly combine with the 3′-UTR of Nrf2 mRNA and reduce the level of Nrf2 and its downstream target genes. Intracerebroventricular injection of miR-93 antagomir decreased cerebral infarct volume, neuronal apoptosis, and oxidative stress after transient middle cerebral artery occlusion (MCAO), providing an attractive prospect for miR-93 as a therapeutic target for ischemic stroke 96.

### Myocardial IRI

**Figure 5:**
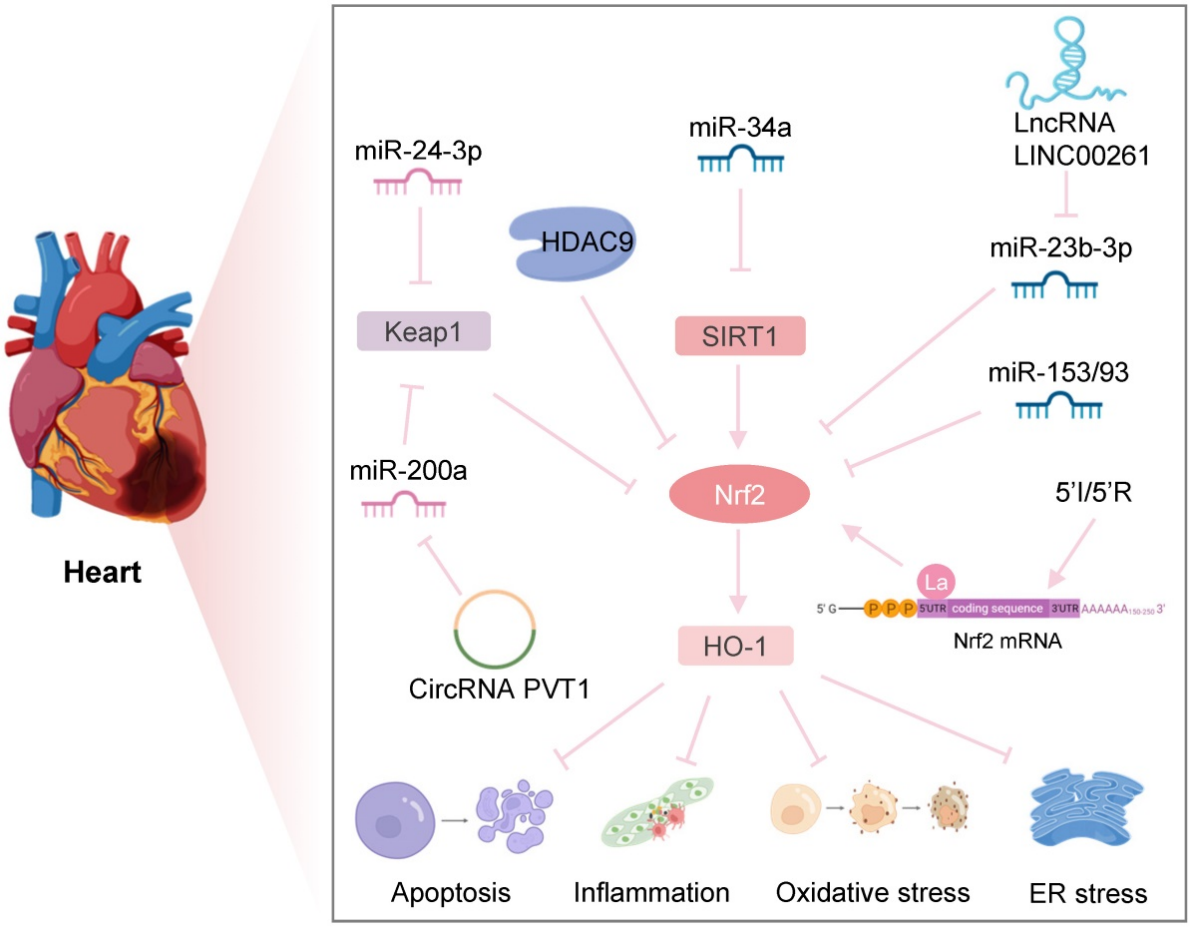
** Nrf2-related epigenetic modifications in myocardial IRI.** Downregulation of HDAC9 regulates hypoxia-induced cardiomyocyte apoptosis by activating the Nrf2/HO-1 pathway. Inhibition of miR-34a expression increases the SIRT1/Nrf2/HO-1 pathway, protecting against ER stress and apoptosis after myocardial I/R. MiR-24-3p reduces I/R-induced apoptosis of cardiomyocytes by reducing Keap1 expression. CircRNA PVT1 alleviates cardiac I/R damage by inhibiting the miR-200a/Keap1/Nrf2 pathway. lncRNA LINC002261 inhibits I/R-induced myocardial apoptosis by restraining the miR-23b-3p/Nrf2 pathway. Upregulation of miR-153 and miR-93 reduces Nrf2 expression to increase I/R-induced apoptosis of myocardial cells. Transient ischemia and reperfusion (5'I/5'R) preadaptation increase the La protein binding to the 5'UTR of Nrf2, exerting protective effects on myocardial IRI.

In an era of increasing reperfusion technology and availability, IRI has become a predominant issue in interventional cardiology 97, 98. After myocardial I/R, multiple factors, including oxidative stress, mitochondrial dysfunction, and cell death, can lead to myocardial damage, resulting in myocardial shock, arrhythmia, increased infarct size, and heart failure. Therefore, it is necessary to study the underlying IRI mechanisms to improve the prognosis of patients with myocardial ischemia (**Figure 5**).

HDAC9 is a class II HDAC family member that is abundantly expressed in the brain and cardiac muscle and is involved in the epigenetic regulation of DNA transcription. A recent study found that Nrf2 mRNA and protein levels were significantly reduced in contrast to the marked upregulation of HDAC9 expression in the *in vitro* and *in vivo* models of myocardial infarction. Down-regulation of HDAC9 regulated cardiomyocyte proliferation, apoptosis, and myogenesis by activating the Nrf2/Keap1/HO-1 pathway, thus improving hypoxia-induced cardiomyocyte injury and cardiac function 99. Interestingly, a genome-wide association study indicated that HDAC9 played a critical role in the development of ischemic stroke, and its variants could increase the risk of ischemic stroke by promoting carotid atherosclerosis 100. These studies suggested that HDAC9 might be a new target for diagnosing and treating ischemic diseases.

A study showed that curcumin treatment exerted an anti-oxidative stress effect by reversing the epigenetic silencing of Nrf2 during prostate tumor development in TRAMP mice. This effect was achieved by curcumin acting as a hypomethylating agent by altering the methylation level of CpG islands in the Nrf2 gene promoter region 12. Li and colleagues discovered a new antioxidant, novel curcumin analog 14p, which could ameliorate oxidative stress and limit myocardial IRI by activating Nrf2 101. However, the specific mechanism by which curcumin analog 14p increases Nrf2 expression and reduces myocardial IRI through demethylation of Nrf2 remains to be elucidated.

In a mouse model of myocardial I/R, Xu et al. observed that overexpression of SIRT1 could significantly reduce Nrf2 acetylation and upregulate the downstream signaling pathway of Nrf2, considerably improving cardiac oxidative stress, cardiac function, and infarct size 102. Another study showed that miR-34a expression was significantly upregulated after myocardial I/R, and inhibition of miR-34a could reduce myocardial injury by eliminating SIRT1 inhibition, reducing apoptosis and infarct size, and improving left ventricular dysfunction 103.

Dang et al. found that inhibition of SETD7 reduced hypoxia/reoxygenation (H/R)-induced cardiomyocyte damage by downregulating Keap1 and promoting Nrf2-mediated antioxidant signaling 104. Interestingly, in diabetic retinopathy development, SETD7 knockout inhibited Keap1 expression by preventing lysine 4 methylation on histone H3 (H3K4), stimulating protein-1 (Sp1) binding to the Keap1 promoter, and promoting Nrf2 expression 105. Furthermore, another study on a human bronchial epithelial cell line (Beas-2B) indicated that SETD7 might directly methylate lysine residues in *Nfe2l2* to alter the interaction between Nrf2 and Keap1, reducing overall Nrf2 protein levels. In addition, in SETD7 knockout cells, increased Nrf2 protein might offer a positive feedback mechanism for its own transcription by binding to the ARE region in the *Nfe2l2* promoter, resulting in a concurrent increase in Nrf2 mRNA levels 106. Therefore, Keap1/Nrf2 epigenetic modifications are expected to provide a new approach for exploring the specific mechanism by which SETD7 regulates H/R-induced cardiomyocyte injury. In another study by Xu et al., cycles of brief ischemia and reperfusion (5′I/5′R) could increase Nrf2 expression and protect mice against myocardial IRI. Specifically, cycles of 5′I/5′R or oxidants increased the binding of the La protein to the Nrf2 5′ UTR, promoting de novo Nrf2 protein translation 107.

Noncoding RNAs have been shown to participate in myocardial IRI, and numerous studies have focused on the function of noncoding RNAs on Nrf2 in developing myocardial IRI. Hou et al. found that in a rat model of I/R, inhibition of miR-153 could activate Nrf2 expression and reduce cardiomyocyte apoptosis, attenuating the inflammatory response and oxidative stress 108. Another study reported that in hypoxic cardiomyocytes, overexpression of miR-200a significantly reduced Keap1, increased nuclear translocation of Nrf2, and the expression of downstream antioxidant enzyme genes, ameliorating cell apoptosis and ROS production and facilitating cardiomyocyte viability. Bioinformatic analysis and a dual-luciferase reporter assay found that miR-200a could bind to the 3'-UTR of Keap1 and inhibited the expression of Keap1 at the post-transcriptional level 109. Moreover, studies have reported that circular RNA PVT1 limited the expression of miR-200a as a composite endogenous RNA in rat myocardial tissue. Circular RNA PVT1 silencing significantly alleviated apoptosis induced by myocardial IRI by increasing the expression of miR-200a 110. These findings shed light on the potential of the circular RNA PVT1/miR-200a/Keap1/Nrf2 axis as a treatment strategy for myocardial IRI.

After OGD/R in cardiomyocytes, elevated miR-93 expression increased apoptosis of cardiomyocytes by targeting inhibitory Nrf2 111. MiR-24-3p is a tumor suppressor that modulates various tumors. Recent studies have demonstrated that the increased expression of miR-24-3p reduces IRI-induced apoptosis of cardiomyocytes *in vitro* by targeting inhibition of Keap1, leading to the activation of Nrf2 signaling 112. Zhang et al. discovered that in I/R myocardial tissue and H/R cardiomyocytes, lncRNA LINC00261 expression was downregulated, while overexpression of LINC00261 reduced the H/R-induced apoptosis of cardiomyocytes. Further evidence suggested that LINC00261 upregulated Nrf2 expression in cardiomyocytes by restraining miR-23b-3p and consequently reducing H/R-induced apoptosis in cardiomyocytes 113.

### Liver IRI

**Figure 6:**
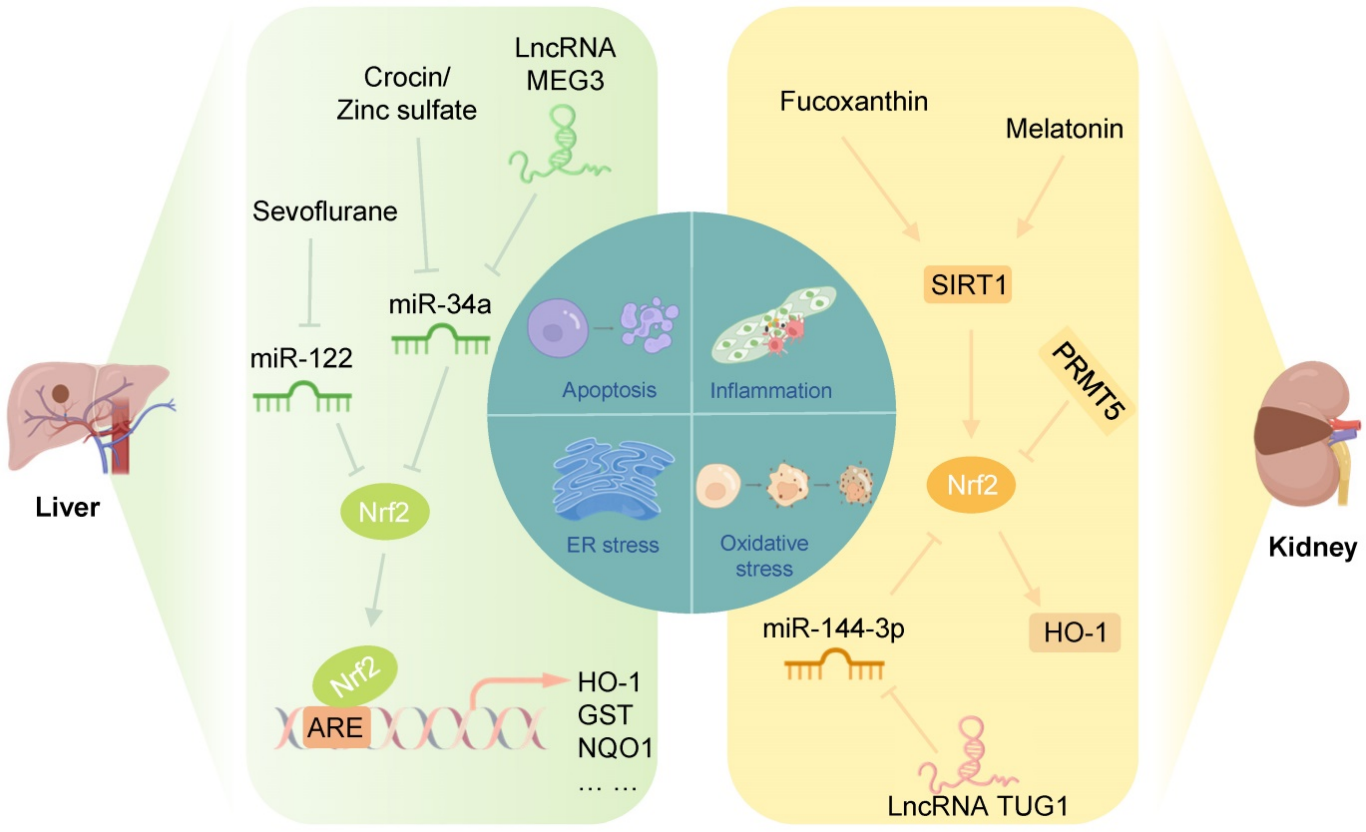
** Nrf2-related epigenetic modifications in liver and renal IRI:** Sevoflurane alleviates the inhibition of Nrf2 by miR-122, inhibiting I/R-induced oxidative stress and inflammation of cardiomyocytes. Crocin, zinc sulfate, and lncRNA MEG3 could promote the Nrf2/ARE pathway by inhibiting miR-34a to improve oxidative stress in liver IRI. Fucoxanthin and melatonin might alleviate apoptosis induced by renal IRI through the SIRT1/Nrf2/HO-1 pathway. PRMT5 promotes oxidative stress and pyroptosis by inhibiting the Nrf2/HO-1 pathway to aggravate renal IRI. The lncRNA TUG1 inhibits renal I/R-induced oxidative stress and apoptosis by suppressing the miR-144-3p/Nrf2 pathway.

Liver IRI is one of the main causes of irreversible injury during liver transplantation and hypovolemic shock. Treatment of hepatic IRI involves multiple aspects, involving genetics, pharmacology, and surgical procedures. This section will focus on the latest treatment methods for hepatic IRI in the context of regulating Nrf2-related epigenetic modifications (**Figure 6**).

Studies have shown that in a rat model of hepatic I/R, crocin and zinc sulfate could promote Nrf2 expression in the liver and reduce the miR-34a level, improving the antioxidant capacity of the liver. Notably, serum miR-34a levels were negatively correlated with Nrf2 levels in rats in the hepatic I/R group 114, 115. Furthermore, Huang et al. showed that miR-34a inhibited the activity of Nrf2 and its ARE targets in the liver in a NaHS-preconditioned young rat model of hepatic I/R 116. These results suggested that inhibition of miR-34a protects against hepatic IRI by increasing the translation of Nrf2 and its downstream antioxidant genes. Another study showed that the lncRNA MEG3, as a ceRNA for miR-34a, protected liver cells from I/R damage by downregulating the expression of miR-34a and upregulating the expression of Nrf2 117.

MiR-122 is a primary liver-specific miRNA that significantly modulates liver development, liver function, and hepatocyte growth 118. The level of circulating miR-122 was markedly increased during liver IRI and positively related to the activity of liver function indicators, such as aspartate aminotransferase (AST), alanine aminotransferase (ALT), and lactate dehydrogenase (LDH), suggesting that miR-122 could be a key molecule for liver I/R therapy 119. A recent study demonstrated that sevoflurane promoted Nrf2 expression in the I/R liver tissue and reduced miR-122 expression, ameliorating I/R-induced oxidative stress, inflammatory responses, and apoptosis in the liver tissue 120. Interestingly, Nrf2 has been identified as a potential target of miR-122 in acute liver injury 121.

### Renal IRI

In the clinical setting, IRI causes acute kidney injury, adverse outcomes after transplantation, and patient morbidity and mortality 122. After renal I/R, elevated production of ROS in damaged tissues is a crucial cause of tubular epithelial cell death and renal injury 123. When ROS generation surpasses the antioxidant ability, further tissue damage and apoptosis are induced 124. Therefore, reducing oxidative stress during renal I/R is crucial for treating renal ischemia. This section will examine the effect of Keap1/Nrf2 on renal IRI and epigenetic modifications of Keap1/Nrf2 to explore new therapeutic targets (**Figure 6**).

In a mouse model of IRI and a human renal proximal tubular epithelial cell line (HK-2) H/R injury model, the marine carotenoid fucoxanthin inhibited oxidative stress-induced apoptosis through the SIRT1/Nrf2/HO-1 signaling pathway, and its mechanism was likely associated with Nrf2 nuclear translocation 125. Shi et al. found that melatonin attenuated acute renal IRI in diabetic rats by activating the SIRT1/Nrf2/HO-1 signaling pathway 126. Also, activation of SIRT1 could stabilize the transcription factor Nrf2 through deacetylation 102, 127. Future studies should focus on whether SIRT1 can improve renal IRI by regulating promoted by SIRT1 Nrf2 deacetylation. Protein arginine methylation transferase 5 (PRMT5), which regulates arginine methylation and participates in the modulation of epigenetics, aggravated renal IRI by promoting oxidative stress and pyroptosis, inhibiting the Nrf2/HO-1 pathway, and suppressing the proliferation of tubular epithelium 128.

Zhao et al. demonstrated that in mouse renal IRI and mouse renal tubular epithelial cell (TCMK cell) H/R injury models, lncRNA TUG1 expression was increased, while that of miR-144-3p decreased significantly. Mechanistic experiments clarified that miR-144-3p could target and inhibit Nrf2 expression. Furthermore, lncRNA TUG1 could promote Nrf2 expression by sponging miR-144-3p and improving oxidative stress damage and apoptosis of cells and tissues 129. In summary, the lncRNA TUG1/miR-144-3p/Nrf2 axis provides a new therapeutic approach for renal IRI.

## Pharmacological targeting of epigenetic modifications of Nrf2

Currently, natural compounds extracted from fruits, vegetables, tea, and medicinal plants are applied to treat various diseases, such as cancer and cardiovascular, neurological, and kidney diseases. These phytochemicals can modulate Nrf2-related epigenetic modifications and overcome oxidative stress at nontoxic concentrations, providing new approaches for the pharmacotherapy of diseases 130-133. The protective effect of Nrf2 agonists has been validated in preclinical models and human clinical trials. The regulation of epigenetic modifications of Nrf2 by phytochemicals is an emerging field that will open up avenues for exploring new strategies to target Nrf2 signaling and provide new ideas for treating ischemic diseases and cancer. This section will summarize recent advances in regulating Nrf2 signaling by various phytochemicals targeting DNA methylation, histone modifications, and noncoding RNAs.

### SIRT1 agonists

As a deacetylase, SIRT1 can regulate gene expression through histone deacetylation and play a key role in preventing oxidative stress and inflammation. For example, SIRT1 could enhance Nrf2 expression through the deacetylation of Nrf2 134. Yang et al. found that quercetin significantly reduced cerebral infarct volume and ROS generation via the SIRT1/Nrf2/HO-1 signaling pathway and exerted a neuroprotective effect on cerebral IRI, which could be reversed by the SIRT1 selective inhibitor EX527 135. In addition, studies have demonstrated that HG could aggravate OGD/R-induced apoptosis, the inflammatory response, and oxidative stress in HT22 hippocampal neurons, which could be alleviated by isorhamnetin. Further studies showed that the isorhamnetin effect was exerted by increasing AKT expression, which activated the SIRT1/Nrf2/HO-1 signaling pathway 136. Isorhamnetin also has positive effects on H/R-stimulated H9C2 cells, ameliorating apoptosis and oxidative stress in H9C2 cells by promoting SIRT1/Nrf2/HO-1-mediated antioxidant signaling 137. Honokiol has been shown to improve myocardial IRI in diabetic rats by facilitating SIRT1 and increasing Nrf2 expression to reduce oxidative stress and apoptosis 138. It was also reported that crocin, a water-soluble carotenoid, may protect cardiomyocytes from endoplasmic reticulum stress and IRI-induced apoptosis by regulating the miR-34a/SIRT1/Nrf2 pathway 139. Furthermore, in rat liver I/R, total flavonoids (TFs) from Rosa laevigata Michx fruit could significantly increase the SIRI1 level in the liver tissue, activating Nrf2 expression and alleviating IRI-induced oxidative stress injury 140.

### Resveratrol

Resveratrol (trans-3,5,400-trihydroxystibene, RV) is a naturally occurring polyphenolic compound in daily diets and some herbal medicines and has been proven to have protective effects against cardiovascular diseases, cancer, and aging 141. In cerebral, myocardial, and renal IRI, RV has been shown to reduce oxidative stress and inflammatory responses by upregulating Nrf2 expression 142-145. Interestingly, recent research has suggested that epigenetic changes regulated by RV are associated with some pathological processes. For instance, in nonalcoholic fatty liver disease (NAFLD), RV treatment could downregulate Nrf2 promoter methylation by decreasing the expression of DNMTs, facilitating Nrf2 transcription, and attenuating oxidative stress 58. However, the epigenetic regulation of Nrf2 by RV in IRI injury has not been reported thus far, identifying an area that needs to be addressed.

### Sulforaphane

Sulforaphane (SFN), a natural and well-studied isothiocyanate and a plant-derived chemoprotective ingredient, is extracted from cruciferous vegetables such as broccoli, cauliflower, cabbage, and kale 146. It is a strong activator of Nrf2 that can exert chemotherapeutic effects by regulating oxidative stress, inflammation, cell proliferation, differentiation, and apoptosis. Studies have shown that SFN can protect the brain, liver, heart, and kidney from IRI injury by increasing Nrf2 expression 147-150. However, the specific mechanism by which SFN regulates Nrf2 expression has not been well explored. Epigenetic modifications provide a new possibility to understand how SFN regulates Nrf2. In studies of Alzheimer's disease, skin cancer, prostate cancer, colon cancer and angiotensin II-induced cardiomyopathy, SFN has been found to exert protective effects through epigenetic modifications. Specifically, SFN reduced methylation of the first 15 CpG islands on the Nrf2 gene promoter, which was associated with reduced DNMT levels, and promoted the enrichment of acetylated histone H3 in the Nrf2 promoter region by inhibiting the expression and activity of HDAC 151-155.

### Ursolic acid

Ursolic acid (UA), a representative pentacyclic triterpenoid obtained from berries and the peels of pears, apples, plums, and other fruits, reduces ROS toxicity and enhances antioxidant enzyme activity 156. Notably, many current studies have revealed that UA can exert a significant effect in treating diseases by modulating the expression of epigenetic modification-related enzymes, such as DNMTs and HDACs 157, 158. As a classic anti-inflammatory and antioxidant molecule, Nrf2 has been shown to be a key target for UA. For example, UA protected the mouse brain against cerebral ischemic injury through the Nrf2 pathway 159. Another study showed that UA treatment increased SETD7 expression in LNCaP cells and H3K4me1 enrichment at the Nrf2 promoter, enhancing Nrf2 expression and protecting DNA from oxidative damage 160. In contrast, SETD7 knockdown ameliorated oxidative stress induced by cerebral IRI by increasing Nrf2 transcriptional activity 90. Furthermore, SETD7 inhibition could reduce Keap1 expression in retinal endothelial cells by preventing H3K4 methylation, thereby increasing Nrf2 expression and reducing apoptosis and oxidative stress damage 105. It is possible that UA can regulate Nrf2 by inducing SETD7 expression, which can regulate Nrf2 expression through epigenetic modifications and reduce IRI damage.

### Luteolin

Luteolin (3′,4′,5,7-tetrahydroxyflavone, LUT), a natural flavone compound extracted from Chinese herbs and vegetables, has many biological benefits, such as anti-inflammatory, antioxidant, cardioprotective, and neuroprotective effects 161-163. A recent study found that LUT could reverse the elevated expression of miR-320 and Nrf2, increasing antioxidant enzymes and reducing oxidative stress in renal I/R 164. In contrast, Zhu et al. found that the miR-320 expression was downregulated in myocardial IRI. Inhibition of miR-320 could enhance the antioxidant capability of the myocardium and reduce apoptosis and IRI by increasing Nrf2 expression 165. The differences in miR-320 expression in the kidney and heart may be due to the tissue-specific expression of miRNAs. The epigenetic regulation of Nrf2 by LUTs has been extensively studied in cancers. For example, Zuo et al. found that in LUT-treated HCT116 and HT29 colon cancer cells, LUT inhibits the protein level and enzymatic activity of DNMT1, DNMT3a, and DNMT3b in a dose-dependent manner. Then, the demethylation of the Nrf2 promoter leads to the activation of Nrf2 and its downstream genes, reduces ROS levels and DNA damage, and inhibits cancer cell proliferation. In addition, the protein and enzymatic activities of HDACs in HCT116 cells also decreased after LUT treatment. Interestingly, subsequent studies reported that in HT-29 and SNU-407 cells, LUT could enhance the transcription activator TET DNA in addition to repressing the expression of the transcriptional repressor DNMTs. It also promotes the combining of TET1 with the Nrf2 promoter, significantly reduces DNA methylation in the Nrf2 promoter region of colon cancer cells, and upregulates the expression of Nrf2, thereby inducing apoptosis of colon cancer cells. Taken together, these studies demonstrate that LUT plays a series of biological roles by increasing the expression of Nrf2 through epigenetic modifications of Nrf2. Unfortunately, the epigenetic modifications of Nrf2 by LUT are still concentrated in the field of tumors, and its role in other diseases needs to be further explored in the future 166, 167. These studies demonstrate that LUT plays a series of biological roles by increasing the expression of Nrf2 through epigenetic modifications of Nrf2, which provides a new research direction for treatment of IRI by LUT.

### Curcumin

Curcumin, also called diferuloylmethane, is a yellow chemical compound generally isolated from the rhizome of turmeric 168. Curcumin has antioxidant, antibacterial, anti-inflammatory, and anticancer properties and can also act as an epigenetic regulator for Nrf2 and its downstream genes.

Li et al. reported that curcumin could reduce neurological dysfunction, cerebral infarct volume, cerebral edema, and blood-brain barrier disorder after cerebral IRI. The mechanism might be associated with the upregulation of Nrf2 expression by curcumin 169. Furthermore, dimethyl fumarate and curcumin combination treatment could significantly reduce serum ALT and AST activities caused by liver I/R and improve liver histopathology. The combined treatment of dimethyl fumarate and curcumin exerted its effect by activating the Nrf2/HO-1 signaling pathway and reducing inflammatory markers (TNF-α, IL-1β, Il-6, and iNOS) 170. It has been reported that C66, a novel analog of curcumin with extremely high bioavailability, increased miR-200a expression in kidneys of diabetic mice, decreased Keap1 at the transcriptional and translational levels, induced Nrf2 function, and ameliorated diabetes-induced oxidative stress damage 171. Similarly, Sun et al. demonstrated that the expression of miR-200a was significantly downregulated in ischemic myocardium and hypoxic cardiomyocytes. Overexpression of miR-200a could substantially increase the nuclear translocation of Nrf2 and expression of downstream antioxidant enzyme genes and alleviate hypoxia-induced oxidative stress injury related to the interaction of miR-200a with the 3'-UTR of Keap1 109. In addition, studies have shown that the occurrence of prostate cancer is related to the hypermethylation of the Nrf2 promoter region and the decreased expression of Nrf2 and its downstream detoxification and antioxidant genes. In murine prostate cancer TRAMP C1 cells, human prostate cancer LnCap cells and human colon cancer HT29 cells, curcumin exerts a preventive effect on cancer by reducing methylation of CpG islands of Nrf2 and activating its expression 12, 172, 173.

### Sodium butyrate

Sodium butyrate (NaB) is a sodium salt metabolite of short-chain fatty acids (SCFAs) produced by microorganisms that degrade dietary fibers in the colonic lumen 174. SCFAs exert a crucial role in preserving gut health, enter the systemic circulation, and directly affect the metabolism and function of peripheral tissues 175. As an HDAC inhibitor, first reported in 1978 176, NaB has shown great potential in oxidative stress, immunomodulation, and cancer prevention and treatment. Recently, NaB has been reported to function as an Nrf2 agonist 177, epigenetic modifications of which play a role in many diseases.

Dong et al. reported that in diabetic mice, NaB treatment ameliorated diabetes-induced renal oxidative damage, apoptosis, inflammation, fibrosis, pathological changes, and renal dysfunction by enhancing Nrf2 and its downstream targets HO-1 and NQO1. Interestingly, the nuclear translocation of Nrf2 or Keap1 expression inhibited HDAC activity, suggesting that NaB promoted the binding of transcription factors to the Nrf2 gene promoter by depressing HDAC activity to activate Nrf2 at the transcriptional level 178. However, sodium butyrate (SB) treatment could activate Nrf2 and downstream gene transcription by increasing hippocampus HDAC1,2 mRNA and histone H4 acetylation levels in chronic cerebral hypoperfusion rats, thereby protecting cognitive function. This contradictory expression may be due to the following reason. The mechanism of action of HDAC inhibitors mainly depends on the interaction of the enzyme and the inhibitor with zinc in the active site. Therefore, insufficient SB-induced enzymatic activity results in compensatory transcription of mRNA 177. Similarly, in HG-treated aortic endothelial cells, NaB inhibited HDAC activity. It increased the occupancy of the transcription factor aryl hydrocarbon receptor (AHR) and the co-activator P300, a histone acetylase, at the Nrf2 gene promoter, resulting in increased Nrf2 transcription to exert a protective role against oxidative stress and inflammation 179. In contrast, in a non-transformed small intestine epithelial IEC-6 cell line, NaB increased the expression of Nrf2 at the gene level and its nuclear translocation 180. These contradictory results could be explained due to different cell types and processing methods used. Also, in a high-fat diet-induced rat model, NaB activated the transcription of Nrf2 and its downstream antioxidant enzymes, ameliorating high-fat diet-induced weight gain, oxidative stress, and insulin resistance. The upregulation of Nrf2 expression in NaB-treated rat livers was associated with a decrease in HDAC1 protein expression and an increase in histone H3 acetyl K9 (H3K9ac) modification on the Nrf2 promoter 181.

## Conclusions and Perspectives

Oxidative stress and inflammation induced by I/R aggravate organ function damage; therefore, preventing and reducing ROS production and inflammatory damage is essential to improve and preserve organ function in IRI. As a major antioxidant factor, Nrf2 protects normal cells from damage by enhancing their antioxidant and detoxification capabilities. Theoretically, during cancer development and progression, Nrf2 could have two functions. Prior to tumorigenesis, Nrf2 could play a crucial role in combating oxidative stress and preventing malignant transformation. After tumor formation, malignantly transformed cells could utilize the Nrf2 defense system to impede cancer cell growth, spread, and resistance to therapy 182, 183. However, the two-faced nature of Nrf2 in IRI has not yet been reported, emphasizing the need for more exploration in this field.

In recent years, the regulatory effect of epigenetic modifications on Keap1/Nrf2 in various diseases has been elucidated. This review focuses on the specific mechanisms by which methylation/demethylation of CpGs in the promoter region of Keap1/Nrf2, methylation/demethylation and acetylation/deacetylation of histones, or miRNAs regulate Keap1/Nrf2 expression in I/R diseases.

The role of epigenetic modifications associated with Keap1/Nrf2 during I/R remains to be better understood. With the identification of endogenous imprinted genes, genomic imprinting became widely recognized as an epigenetic mechanism in which the expression pattern of a parental allele influences phenotypic expression 184. Besides the canonical genomic imprinting mediated by allelic DNA methylation, recent studies have shown that maternal H3K27me3 can lead to imprinting unrelated to DNA methylation, termed “non-canonical imprinting”. Abnormal expression of these genes has been found in genetic diseases, cancer, and autoimmune diseases 185. Future research on genomic imprinting may better explain individual differences in resistance to IRI and provide new ideas for developing therapeutic drugs for IRI. In 2013, Young et al. discovered a new class of gene regulatory elements called "super-enhancers," characterized by clusters of adjacent large enhancers that drive the specific expression of genes 186. It has been demonstrated that transcriptional programs can be regulated by epigenetic alterations in super-enhancers, which play an important role in maintaining cancer cell properties 187. Furthermore, a recent study of renal IRI demonstrated that renal injury results in genome-wide alterations in the enhancer and super-enhancer repertoire. Specifically, 216 injury-associated super-enhancers, 164 super-enhancers lost during injury, and 385 SHARED super-enhancers were identified by using the ROSE algorithm, which were present in kidney samples at baseline and IRI day 2 sample. Most of these super-enhancers can play important roles in renal epithelial cells by regulating gene expression 188. Although the current research on super-enhancers in IRI is limited, a better understanding of the regulation of Nrf2 transcription with the possible involvement of super-enhancers would have far-reaching significance.

We have also reviewed compounds that regulate the Keap1/Nrf2 pathway through epigenetic modifications. However, most of the current research on the epigenetic modification effects of Nrf2 agonists has focused on cancer treatment, and few studies have elaborated on the mechanism of these drugs and their potential in IRI, indicating the need for mechanistic studies in this field.

Owing to the complex pathophysiology of IRI, there is currently no effective approach to counterbalance this injury. Therefore, studying the function of Nrf2 in IRI, especially the role of Nrf2-related epigenetic modifications, will provide a new approach to treating I/R-related diseases.

## Figures and Tables

**Table 1:** Pharmacological targeting of epigenetic modifications of Nrf2

Drugs	Cell/Animal type	Epigenetic mechanism	Molecular targets	Ref.
Quercetin	Transient middle cerebral artery occlusion (tMCAO) rats	Decreased histone acetylation	Increase expression of SIRT1	135
Isorhamnetin	HT22 cells	Decreased histone acetylation	Increase expression of AKT and SIRT1	136
	H9C2 cells	Decreased histone acetylation	Increase expression of SIRT1	137
Honokiol	Type 1 diabetes (T1D) rats	Decreased histone acetylation	Increase expression of SIRT1	138
Crocin	Myocardial I/R-induced primary cardiomyocytes and mouse	Reduced expression of miR-34a	Increase expression of SIRT1 and Nrf2	139
Total flavonoids from Rosa laevigata Michx fruit	Hepatic I/R-induced rats	Decreased histone acetylation	Increase expression of SIRT1	140
Resveratrol	HepG2 cells	Reduce methylation of *Nfe2l2*	Suppressed expression of DNMTs	58
Sulforaphane	N2a/APPswe cells	Reduce methylation of *Nfe2l2*	Suppressed expression of DNMTs	151
	JB6 P_+_ cells	Reduce methylation of *Nfe2l2*	Suppressed expression of DNMTs; decreased HDAC1, 2, 3, 4; reduced activities of HDACs	152
	TRAMP C1 cells	Reduce methylation of *Nfe2l2*	Suppressed expression of DNMT1, 3a; decreased HDAC1,4,5,7	153, 154
	human Caco-2 cells	Reduce methylation of *Nfe2l2*	Suppressed expression and activities of DNMT1 protein	154
	C57/BL mice	Reduce methylation of *Nfe2l2*; promoted Ac- H3 accumulation in the Nrf2 promoter region	Suppressed expression and activities of DNMTs; decreased expression and activities of HDACs	155
Ursolic acid	JB6 P_+_ cells	Reduce methylation of *Nfe2l2*	Suppressed expression of DNMT1, 3a; decreased expression of HDAC1, 2, 3, 6, 7, 8; inhibit activities of HDACs	158
	LNCaP cells	Increased H3K4me1 enrichment at the Nrf2 promoter	Induced expression of Setd7	160
Luteolin	Renal I/R-induced rats	Reduced expression of miR-320	Decrease expression of Nrf2	164
	HCT116 cells	Reduce methylation of *Nfe2l2*	Suppressed expression of DNMT1, 3a, 3b; decreased expression and activities of HDACs	166
	HT-29/SNU-407 cells	Reduce methylation of *Nfe2l2*	Suppressed expression of DNMTs; increased TETs	167
Curcumin	TRAMP C1/LnCap/HT29 cells	Reduce methylation of *Nfe2l2*	Decreased activities of DNMTs	12, 172, 173
C66	C57BL/6J mice	Increase miR-200a targeting 3'UTR of Keap1	Increased expression of miR-200a	171
Sodium butyrate	Chronic cerebral hypoperfusion rats	Increasing histone H4 acetylation levels	Increasing HDAC1,2 mRNA levels	177
	C57BL/6 mice	Increase occupancy of the AHR and P300 at the Nrf2 gene promoter; inhibit HDACs activity		179
	Sprague-Dawley rats	Increased H3K9ac at the Nrf2 promoter	Decreased expression of HDAC1	181

## Abbreviations

AREs: antioxidant response elements
Cul3: Cullin-3
circRNAs: circular RNAs
DNMTs: DNA methyltransferases
GR: glucocorticoid receptor
HATs: histone acetyltransferases
HDACs: histone deacetylases
HMT: histone methyltransferase
H3K27ac: acetylation of histone H3 lysine 27
H3K27me3: trimethylation of histone H3 lysine 27
HOTAIR: Hox transcript antisense intergenic RNA
HO-1: heme oxygenase-1
HG: high glucose
H/R: hypoxia/reoxygenation
H3K4: lysine 4 on histone H3
IRI: ischemic-reperfusion injury
I/R: ischemia‒reperfusion
Keap1: Kelch-like ECH-associated protein 1
lncRNAs: long noncoding RNAs
miRNAs: microRNAs
Nrf2: nuclear factor erythroid 2-related factor 2
NQO1: NAD(P)H quinone oxidoreductase 1
Neh: Nrf2-ECH homology
NFAT5: nuclear factor of activated T cells 5
NaB: Sodium butyrate
OGD/R: oxygen-glucose deprivation/restoration
OGSD/R: oxygen-glucose-serum deprivation/restoration
ROS: reactive oxygen species
RV: resveratrol
SIRT1: silencing information regulator 2 related enzyme 1
SETD7: SET domain-containing lysine methyltransferase 7
sMAF: musculoaponeurotic fibrosarcoma
TET: ten-eleven translocation
TPA: 12-O-tetradecanoylphorbol-13-acetate
TSA: trichostatin A
UTRs: untranslated regions
